# Barriers to Mental Health Help-Seeking Amongst Refugee Men

**DOI:** 10.3390/ijerph16152634

**Published:** 2019-07-24

**Authors:** Yulisha Byrow, Rosanna Pajak, Tadgh McMahon, Amitabh Rajouria, Angela Nickerson

**Affiliations:** 1 School of Psychology, University of New South Wales, Sydney, NSW 2052, Australia; 2 Settlement Services International, Sydney, NSW 2131, Australia; 3 Faculty of Medicine, Nursing and Health Sciences, Flinders University, Adelaide, SA 5042, Australia

**Keywords:** refugees, help-seeking, trauma, Post-traumatic stress disorder, stigma, visa security

## Abstract

Rates of help-seeking for mental health problems are low amongst refugee communities, despite the high prevalence of PTSD reported amongst these individuals. Research suggests that the key barriers to seeking help for psychological problems include structural barriers (e.g., unstable housing), cultural barriers (e.g., mental health stigma), and barriers specific to refugees and asylum seekers (e.g., visa status). This study examined the effect of structural, cultural and refugee specific barriers on the relationship between PTSD symptom severity and intentions to seek help from professional, social, and community sources. Data was collected from 103 male refugees and asylum seekers with an Arabic-, Farsi-, or Tamil-speaking background. Participants completed measures indexing demographics, trauma exposure, PTSD symptoms, mental health stigma, and help-seeking intentions. Path analyses indicated that PTSD severity was associated with lower help-seeking intentions indirectly via mental health stigma (self-stigma for seeking help and self-stigma for PTSD) and visa security. PTSD severity was also associated with greater help-seeking intentions from community members indirectly via structural barriers. These findings are important to consider when identifying key barriers to mental health help-seeking and developing interventions designed to increase help-seeking for psychological problems, within this group.

## 1. Introduction

There are currently an estimated 70.8 million forcibly displaced persons worldwide, with this number growing each year [1]. Refugees are typically exposed to numerous traumatic events in the context of ongoing conflict and persecution, including witnessing the murder of family or friends, torture, and being seriously injured [2]. Exposure to traumatic events is a strong risk factor for developing psychological disorders [3], and can have serious long-term effects on mental health [4]. Coping with previous trauma within the resettlement country context, which may be characterized by a lack of social support and ongoing stress, places these individuals at increased risk of developing a psychological disorder [5,6]. Accordingly, rates of post-traumatic stress disorder (PTSD), in particular, are disproportionately higher in refugee samples [3,7] than in the general population [8,9]

Despite the high prevalence of PTSD amongst individuals from refugee or asylum-seeking backgrounds, rates of mental health help-seeking for psychological problems are low amongst these communities [10,11,12,13,14,15]. Mental health help-seeking refers to seeking assistance for mental health related problems and incorporates seeking help from both professional and nonprofessional sources [16,17]. In most western resettlement countries, refugees with permanent and secure visas have access to free/or reduced cost specialized psychological services, as well as mainstream mental health services. Findings from the few studies investigating the prevalence of help-seeking amongst refugees with mental illness compared to individuals with mental illness living in the host country suggest that help-seeking is lower amongst refugees [18,19].

Studies to date have identified a number of key barriers to accessing treatment for refugees, comprising structural barriers (e.g., financial strain and unstable housing), cultural barriers (e.g., stigma relating to mental health), and barriers specific to refugees and asylum seekers (e.g., visa status). Previous research has shown that structural barriers exert an especially great influence in low-income communities in Western countries [20] where individuals experience high levels of unmet basic needs including social isolation, unstable housing, and financial strain [21,22,23]. Important structural barriers faced by refugees include not speaking the language of the resettlement country and a lack of knowledge regarding how to access appropriate services [24,25,26,27]. In addition, cultural barriers, such as mental health stigma, have emerged as being salient in influencing help-seeking for refugees. Self-stigma relating to mental health refers to the negative beliefs an individual holds about his or her own psychological symptoms or help-seeking [28]. Given refugees represent a diversity of cultural groups, it may be that certain broader cultural beliefs about mental health could influence individual attitudes towards treatment. While there has been relatively little investigation of mental health stigma in refugee groups, studies that have implemented standardized measures have found that mental health stigma is more prevalent amongst refugee communities compared to Western resettlement communities [29,30,31]. These findings suggest that mental health stigma is likely to significantly impact mental health help-seeking in refugees.

Finally, barriers to mental health help-seeking can also be identified that are specific to the refugee experience. For example, refugees living in resettlement countries differ according to whether they hold secure visas (i.e., permanent protection, citizenship) or insecure visas (e.g., temporary visa, bridging visa). Research suggests that refugees with insecure visas report substantially higher rates of psychological disorders, including PTSD and depression, and poorer quality of life, compared to those with secure visas [6,32,33]. The only study conducted to date that directly investigated the association between visa security and mental health help-seeking found that individuals with a refugee visa status were significantly less likely to seek help for mental health problems compared to those with citizen visa status [29]. It may be the case that individuals with insecure visa status demonstrate reduced help-seeking either because of logistical difficulties (i.e., differences in available access to mental health care for those with secure vs. insecure visas), or difficulty trusting authority figures stemming from negative interactions with immigration officials throughout the asylum process which may compound negative experiences with authority figures in the resettlement country. It is also likely that structural, cultural, and refugee-specific barriers interact; for example, lack of knowledge about the systems in the host country (structural barrier) may increase mental health stigma (cultural barrier), and may be exacerbated by not wanting to disclose psychological symptoms for fear of not getting a permanent visa (refugee-specific barrier).

To date, however, there have been no studies that have concurrently examined the association between each of these types of barriers and mental health help-seeking amongst refugees. The current study is the first to examine the relative associations between structural (financial strain, unstable accommodation), cultural (mental health stigma), and refugee-specific barriers (visa status) with help-seeking intentions in a group of refugee men with varying levels of PTSD symptoms. We predict that greater PTSD symptom severity will be associated with lower help-seeking intentions via higher mental health stigma, insecure visa status, and greater structural barriers.

## 2. Materials and Methods

### 2.1. Participants and Procedure

A total sample of 103 refugee males participated in the current study, which was conducted as part of a larger randomized controlled trial examining the effectiveness of a stigma reduction intervention on refugee men. The data used in the following analyses is drawn from the baseline assessment time-point of the larger study (prior to receiving the stigma reduction intervention). Eleven participants with missing data on independent variables were excluded from the analyses, which lead to a final sample size of 92. Eligible participants were male, aged over 18 years old, were refugees or asylum seekers residing in Australia, had internet access and could complete measures in Arabic, Farsi, or Tamil. At the time of enrolment in the study all participants reported experiencing at least some PTSD symptoms. Participants who were currently seeing a mental health professional or actively suicidal were excluded from this study.

The current study was approved by the University of New South Wales Human Research Ethics Committee (HC15351) and written informed consent was obtained from all participants. Participants were recruited to the larger trial by (i) being referred by caseworkers providing settlement services and (ii) responding to advertisements placed at relevant service provider offices and online. Following this, participants were screened by a telephone interview (with interpreters where necessary) assessing eligibility. Eligible participants completed all measures online in their own language (Arabic, Farsi, or Tamil). Consistent with World Health Organization standards, all self-report measures included in this study were forward and blind back-translated by professional accredited translators fluent in Arabic, Farsi, and Tamil, as well as English [34].

### 2.2. Measures

#### 2.2.1. Exposure to Potentially Traumatic Events (PTEs)

The Harvard Trauma Questionnaire (HTQ) [35] is a 16 item scale that assesses exposure to traumatic events commonly experienced by refugees. Example events assessed included ‘torture’, ‘imprisonment’ and ‘rape or sexual abuse’. Participants indicated, for each event, whether they experienced it themselves, witnessed it happening to others, learned about it happening to a friend/family member, or none of the above. Items endorsed as experienced or witnessed were considered a positive response. These responses were summed to create a total count of exposure to different types of traumatic events.

#### 2.2.2. Post-Traumatic Stress Symptoms

The Post-Traumatic Diagnostic Scale (symptom scale) (PDS) [36] is a 21-item measure of PTSD symptom severity. Participants indicated how often they experience specific PTSD symptoms by responding on a 4-point scale (0—Not at all or only one time; 1—Once a week or less/once in a while; 2—2 to 4 times a week/half the time; 3—5 or more times a week/almost always). Scores were summed to create a total score representing PTSD symptom severity. This measure has previously been used with refugee samples [37] and has demonstrated strong psychometric properties including internal consistency, test–retest reliability, and convergent validity [36]. In the current study, internal consistency of the PDS measure was α = 0.95.

#### 2.2.3. Structural Barriers to Help-Seeking

Potential structural barriers to help-seeking for mental health were measured using an adapted version of the Postmigration Living Difficulties Checklist (PMLDC) [38]. Items indexing structural barriers included “Not being allowed to work”, “Not being able to find work”, “Not being able to access English language training”, “Not enough money to buy food, pay the rent and bills, or buy necessary clothes”, “Difficulty accessing public transport, or not having enough money to use public transport”, “Difficulties obtaining financial assistance from Government or Charities”, and “Difficulties relating to housing (e.g., poor housing conditions, difficulty finding somewhere suitable to live, having to frequently change place of residence).” Participants responded to these items on a 5-point scale (1—Was not a problem/Did not happen; 2—A small problem; 3—A moderately serious problem; 4—A serious problem; 5—A very serious problem). Items rated as at least 3 (moderately serious problem) on this scale, were summed to create a total structural barriers to help-seeking scale. The internal consistency of this measure was good (α = 0.86).

#### 2.2.4. Stigma Associated with Help-Seeking Scale

The Self-Stigma of Seeking Help scale (SSOSH) [39] is a 10-item measure of self-stigma associated with seeking professional psychological help. Participants responded to items (e.g., “It would make me feel inferior to ask a therapist for help”) on a 5-point rating scale (1—Strongly Disagree; 2—Disagree; 3—Neutral; 4—Agree; 5—Strongly Agree). Higher scores represented greater stigma associated with help-seeking. This measure has demonstrated good psychometric properties including internal consistency in previous studies [39] as well as the current study (α = 0.82).

#### 2.2.5. Stigma Associated with PTSD

The Self-Stigma of Depression Scale (SSDS) [40] was adapted to measure the extent to which an individual holds negative beliefs in relation to having symptoms of PTSD (Self-Stigma of PTSD Scale; SSPS). The scale consists of 16 items (e.g., “I would feel embarrassed about seeking professional help for post-traumatic stress”) and comprises four subscales: shame, self-blame, social inadequacy, and help-seeking inhibition. Participants respond to each item on 5-point scale (1—Strongly Disagree; 2—Disagree; 3—Neutral; 4—Agree; 5—Strongly Agree). Items were summed to create total scores, with higher scores representing greater stigma related to PTSD symptoms. The SSDS has demonstrated adequate psychometric properties including test–retest reliability and internal consistency in previous studies [40]. Following procedures outlined in Nickerson et al., (2019) [41] examination of the association between subscales indicated that the self-blame subscale was not significantly correlated with the other subscales and was thus omitted from calculations of the total score. In the current study the internal consistency of the SSPS was 0.91.

#### 2.2.6. Help-Seeking Intentions

The General Help-Seeking Questionnaire (personal and emotional problems subscale) (GHSQ) [42] is a measure of intentions to seek help from informal (e.g., family) and formal sources (e.g., mental health professional (counselor/psychologist)) for a personal or emotional problem. The original version was adapted to more accurately reflect the professionals and community members affiliated with refugee communities, for example, ‘social worker’ was replaced with ‘caseworker’ (the term typically used in Australia to refer to the social worker assigned to support refugees during the resettlement period). The measure contains 12 items to which participants respond on a 7-point scale (1—Extremely unlikely; 2–3—unlikely; 4–5—likely; 6–7—Extremely likely). The mean ratings for items were used to index help-seeking intentions from family and friends (friends, partner, parent, child, other family member), professionals (caseworker, doctor, mental health professional), and community members (religious leader, community leader). Thus, we created a total score of intentions to seek help from different sources (social, professional, and community sources), with higher scores indicating greater intentions to seek help. The GHSQ has previously demonstrated good internal consistency and test–retest reliability [42]. In the current study the overall internal consistency of the GHSQ measure was α = 0.87. The Spearman-Brown coefficient for the 2 item community subscale was acceptable at 0.52. For subscales related to family and friends and professionals the internal consistency was also acceptable (family: α = 0.79; professionals: α = 0.77).

### 2.3. Data Analysis

A path analysis was conducted in MPLUS Version 8 (Muthén & Muthén, Los Angeles, CA, USA) to examine the relationship between PTSD severity and help-seeking intentions (from professional, social, and community sources) via self-stigma related to PTSD, self-stigma related to help-seeking, structural barriers, and visa security [43]. The model controlled for the association between language (Arabic, Farsi, and Tamil), age, trauma exposure, and PTSD symptom severity. Both direct effects and indirect effects were investigated in this model. A maximum likelihood estimator was implemented, which uses other variables in the model to adjust parameters, thus accounting for missing data; and analyses were bootstrapped, using 5000 samples [44,45]. Preliminary analyses examining the effects of PTSD symptom severity on intentions to seek help via self-stigma related to PTSD and self-stigma related to help-seeking, structural barriers, and visa security, while controlling for language (Arabic, Farsi, and Tamil), age, and trauma exposure, showed no significant effects of language or age on PTSD symptom severity. Thus, language and age were not included as covariates in the final model. Model fit was assessed using the following criteria; a nonsignificant chi-square (χ^2^) test, the root mean square error of approximation (RMSEA) < 0.05, Comparative Fit Index (CFI) ≥ 0.90, the Tucker—Lewis Index (TLI) ≥ 0.90, and standardized root mean square residual (SRMR) < 0.05 [46,47].

## 3. Results

### 3.1. Participant Characteristics

Participants were aged between 20 and 64 years with an average age of 40.23 years (*SD* = 9.94). The language composition of the sample was as follows Arabic *n* = 79 (76.7%); Farsi-speaking *n* = 18 (17.5%), and Tamil *n* = 6 (5.8%). Sixty-seven participants (65%) and 25 participants (24.3%) were secure and insecure visa holders, respectively. The sample characteristics and descriptive statistics of variables included in the following analyses are presented in Table 1. The frequency of exposure to PTEs is presented in Table 2. Participants had experienced or witnessed an average of 7.47 different types of PTEs.

### 3.2. Path Analysis

**Figure 1:**
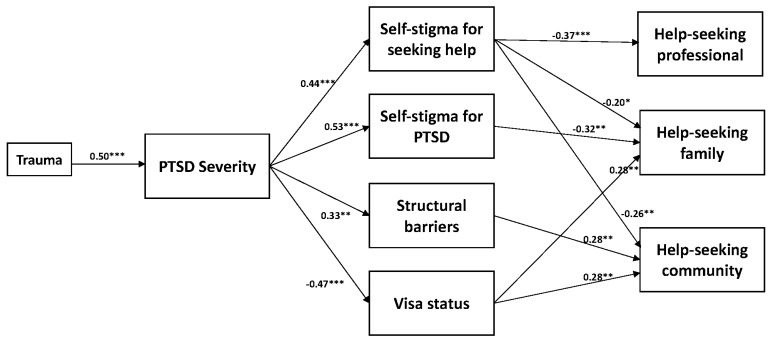
A schematic representing standardized effect sizes in the final reduced model, which examines the effect of PTSD severity on help-seeking via self-stigma for seeking help, self-stigma for PTSD, structural barriers, and visa status. * *p* < 0.05, ** *p* < 0.01, *** *p* < 0.0001.

The initial model examined the indirect effects of PTSD symptom severity on intentions to seek help from family & friends, community members, or professionals for a psychological problem via mental health stigma, structural barriers, and visa security. In addition, we controlled for the effects of trauma exposure on PTSD symptom severity. This model demonstrated adequate model fit (χ^2^ = 13.15, *p* = 0.591; RMSEA < 0.0001; CFI = 1.00; TLI = 1.02; SRMR = 0.05). We then removed nonsignificant pathways to achieve a parsimonious model. The final model demonstrated slightly improved model fit (χ^2^ = 15.22, *p* = 0.708; RMSEA < 0.0001; CFI = 1.00; TLI = 1.03; SRMR = 0.05). Standardized parameter estimates of the final model are displayed in Figure 1.

#### 3.2.1. Direct Effects

Greater severity of PTSD symptoms was significantly associated with self-stigma for PTSD (*β* = 0.53, *p* < 0.0001), self-stigma for seeking help (*β* = 0.44, *p* < 0.0001), structural barriers (*β* = 0.33, *p* = 0.003), and an insecure visa status (*β* = −0.47, *p* = <0.0001). Elevated self-stigma for PTSD was significantly associated with reduced intentions to seek help from family/friends (*β* = −0.32, *p* = 0.001). Elevated self-stigma for seeking help was significantly associated with reduced intentions to seek help from professionals (*β* = −0.37, *p* < 0.0001), family/friends (*β* = −0.20, *p* = 0.040), and community members (*β* = −0.26, *p* = 0.002). Experiencing greater structural barriers was associated with increased intentions to seek help from community members (*β* = 0.28, *p* = 0.001). A secure visa status was associated with increased intentions to seek help from family/friends (*β* = 0.28, *p* = 0.002) and community members (*β* = 0.28, *p* = 0.001). The final model accounted for 35% (*p* < 0.0001) of the variance in help-seeking from family/friends, 20% (*p* = 0.002) from community members, and 14% (*p* = 0.053) from a professional. Trauma exposure and speaking Farsi (compared to Arabic) was associated with higher PTSD severity. Being older was associated with lower PTSD symptoms.

#### 3.2.2. Indirect Effects

There were significant indirect effects of PTSD severity on intentions to seek help from family/friends via self-stigma for PTSD (estimate = −0.17; standard error = 0.05; *p* = 0.001; 95%CI (−0.332, −0.049)); and visa status (estimate = −0.13; standard error = 0.05; *p* = 0.015; 95% CI (−0.286, −0.018)). There was a marginal effect of PTSD severity on intentions to seek help from family/friends via self-stigma for seeking help (estimate = −0.09; standard error = 0.05; *p* = 0.060; 95% CI (−0.233, 0.020)). There was a significant indirect effect of PTSD severity on intentions to seek help from professionals via self-stigma for seeking help (estimate = −0.16; standard error = 0.05; *p* = 0.002; 95% CI (−0.310, −0.040)). There were significant indirect effects of PTSD severity on intentions to seek help from community members via self-stigma for seeking help (estimate = −0.12; standard error = 0.04; *p* = 0.007; 95% CI (−0.234, −0.015)), structural barriers (estimate = 0.09; standard error=0.04; *p*=0.037; 95%CI (0.008, 0.231)), and visa status (estimate = −0.13; standard error = 0.05; *p* = 0.013; 95% CI (−0.288, −0.022)). See Table 3 for a summary of indirect effects.

## 4. Discussion

To our knowledge, this study is the first to examine the relative contributions of mental health stigma, structural barriers, and visa security to intentions to seek help for mental health problems from professionals, social, and community sources, amongst resettled refugees. Findings from the path model suggest that greater PTSD severity was associated with lower help-seeking intentions indirectly via mental health stigma (self-stigma for seeking help and self-stigma for PTSD) and visa security. In contrast, PTSD severity was associated with greater help-seeking intentions from community members indirectly via structural barriers. Specifically, experiencing greater structural barriers was associated with increased intentions to seek help from community members. Accordingly, these results suggest that particular kinds of barriers differentially impact on intentions to seek help from specific sources of support amongst refugee men experiencing symptoms of PTSD.

The current results suggested that self-stigma for seeking help was a more pervasive barrier in terms of its impact on help-seeking, compared to self-stigma for PTSD, affecting intentions to seek help from professionals, community sources, and marginal effects on social sources. Findings from qualitative studies conducted with participants from a refugee background suggest that a lack of trust stemming from persecution experiences [38,48,49,50] and fear of breaches of confidentiality are important concerns for these individuals, which consequently affect help-seeking intentions and behavior [18,51,52,53]. Specifically, concerns about confidentiality could be related to a general fear of authority figures [18], a lack of trust in an interpreter who may be a part of the clients extended social network [51,53], or concern that details, which could compromise the safety of other family members living in the country of origin, will be shared [53]. This is consistent with broader research, with a meta-analysis examining mental health stigma across a broad range of populations, identifying stigma associated with seeking treatment for mental illness as a key type of self-stigma that deterred help-seeking [54]. Overall, the current findings suggest that self-stigma for seeking help is a key barrier for refugees to seek treatment from a range of social, community, and professional sources.

Self-stigma for PTSD was specifically associated with reduced help-seeking from social sources such as family and friends. The concept of self-stigma for PTSD refers to the extent to which an individual perceives the experience of their own PTSD symptoms as negative, embarrassing, or shameful. Previous qualitative [51,55,56,57,58] and quantitative [59] findings suggest that individuals with a refugee background were concerned about eliciting disapproval or being perceived negatively by family members. This may lead to fears that the family unit may be negatively viewed within the community, potentially affecting perceived social opportunities such as marriage prospects, and ultimately eliciting feelings of guilt and shame [51,53]. This may then result in a reduced likelihood of seeking help from informal sources such as family and friends [59]. This is consistent with broader research, with feelings of shame and embarrassment associated with symptoms of mental illness having been shown to be a key barrier to help-seeking across a diverse range of populations, including military, and youth samples [54,60]. Results from this study thus suggest that self-stigma for PTSD is a key barrier to seeking help from social sources for individuals with a refugee background.

The current finding may also be associated with the collectivist backgrounds of the participants. Individuals from collectivist cultures are highly dependent on familial and social relationships and their self-identity is developed within the context of this wider social community [61]. Thus, seeking social support from family and friends is a critical element engrained within culture and identity. Qualitative research with refugees suggests seeking support from social sources commonly occurs and is an important informal help-seeking option [56,62,63]. Ultimately, however, help-seeking is likely to be dependent upon the beliefs held about mental illness by individuals within their social sphere. For example, Papadopoulos, Foster, and Caldwell (2013) [64] suggest that greater collectivism may be associated with higher levels of mental health stigma. Thus, it may be that participants in this study were likely to be concerned about the impact of their mental illness on the family unit, which is likely salient due to their collectivist cultural attributions.

The current findings indicate that an insecure visa status is an additional barrier to seeking help from social and community sources. Several studies have shown that an insecure visa status is associated with poorer mental health and psychosocial adjustment in the resettlement environment [32,65,66]. One possible explanation for this is that those with insecure visas may be more likely to experience family separation (in Australia [67]). Thus, it may be challenging for insecure visa holders to seek support from social and community sources when their social network is greatly reduced. It is interesting that insecure visa status was not a significant barrier to seeking treatment from a professional and may reflect the availability of psychological services in Australia, where both insecure and secure visa holders were entitled to access the same free psychological services at the time of data collection. To our knowledge, this is the first study to empirically investigate how visa insecurity impacts help-seeking intentions for mental illness. The current findings suggest that an insecure visa status directly impacts mental health help-seeking intentions for refugees, indicating that the detrimental effects of an uncertain visa status may extend beyond psychological symptom severity [6,32,33] and could also impact on informal help-seeking for mental health problems. 

The finding that structural barriers were linked to greater intentions to seek help from the community for mental health problems was unexpected. Previous qualitative [68] and quantitative studies [26,27], however, suggest that structural barriers, including financial strain and unstable housing, are important barriers to help-seeking from formal support services for refugees. One possible explanation for the current findings relates to the design of this study. This study is the first, to our knowledge, to concurrently examine the association between structural, cultural, and refugee-related barriers on help-seeking. It is notable that self-stigma for seeking help was negatively associated with accessing support from community members, while structural barriers were positively associated with seeking help from community members. The current findings may reflect the parsing out of different motivations for help-seeking; i.e., the association between stigma and community help-seeking may be more reflective of negative beliefs about seeking help for psychological symptoms which increase with stigma; whereas the association between structural barriers and help-seeking may be reflective of greater need for material assistance. Further research disentangling the complex associations between help-seeking from different formal and informal sources is necessary.

To date, interventions have been developed to target both public and self-stigma within specific samples including for example, college students, specific cultural groups and military personnel in addition to the general population [69,70,71]. Previous reviews of mental health stigma interventions indicate that education- and social contact-based strategies are effective in achieving positive changes in public attitudes and improving knowledge about mental health and help-seeking behaviors, at least in the short-term [69,70,71]. These studies also suggest that findings applicable to a specific group do not necessarily generalize to other groups and that it is critical that mental health stigma interventions are developed with the specific needs of the target group in mind [69,71].

The current findings offer insights into the strategies that could be incorporated into interventions designed to encourage help-seeking for mental health problems amongst refugees. Firstly, a significant implication of the current findings is that mental health stigma is an important target for interventions that aim to reduce barriers and encourage help-seeking amongst refugee men. Incorporating strategies that have been shown to be effective such as social contact may also be effective in changing attitudes and help-seeking behaviors in those with a refugee background. For example, incorporating videos of people sharing their own personal experience of mental illness into interventions has been shown to reduce negative perceptions of those with mental illness, in college students [72]. Given the unique nature of the refugee experience it seems that the strategy of social contact is likely to resonate with these individuals and may be effective in reducing mental health stigma. This is consistent with the findings of a recent study conducted with the same sample, which evaluated the impact of an online intervention for mental health stigma amongst refugee men with PTSD symptoms. This study found that exposure to an online intervention that implemented a variety of strategies including social contact, cognitive reappraisal and psychoeducation led to greater increases in help-seeking behaviors, and smaller increases in self-stigma for seeking help compared to a wait list control group [41]. A second important implication of the current findings is that help-seeking intentions may be different for secure and insecure visa holders, thus interventions to increase help-seeking for mental health may benefit from tailoring to suit an individual’s visa status. These findings, however, are novel and require replication.

The current study has several limitations. The cross-sectional nature of the current study prevents us from drawing conclusions about causality. Thus, further longitudinal studies will be invaluable in confirming the relationship between visa status and help-seeking and will also allow us to assess the longer term impact of visa status on help-seeking. This study focused on self-stigma related to mental health. Public stigma related to mental health, which refers to negative attitudes held by the broader community toward those who seek help for mental health problems, was not examined in this study. However, previous studies have shown that public stigmatization related to mental health can precede self-stigma [73]. Thus, further research investigating the effects of public stigma on help-seeking in refugee communities is necessary and could potentially identify an important target for interventions. Finally, the current study has focused on examining barriers to help-seeking by grouping potential barriers (e.g., financial difficulties and unstable housing) into relevant categories (e.g., structural barriers). Further research delineating the effects of specific barriers to accessing treatment is critical to make specific recommendations for service provision. The current findings, do however, suggest that the high levels of unmet basic needs experienced by refugees, including unstable accommodation and lack of social contact [21,22,23] compel these individuals to seek help from community sources. Thus, it may be worthwhile for service providers to pair mental health education with the delivery of services targeting logistical problems in these communities. Given the preliminary nature of these findings it would be important for future research to deconstruct the relationship between structural barriers and mental health help-seeking further.

The current findings speak to the importance of addressing self-stigma and visa status as important barriers to seeking help (for mental health) for male refugees from Arabic-, Farsi-, and Tamil-speaking backgrounds. The generalisability of these results, however, is subject to certain limitations. For example, the current findings are not necessarily applicable to females from the same communities. Given that several qualitative studies implicate self-stigma as an important barrier to accessing treatment for mental health amongst females, it is imperative that interventions are developed and tailored to suit the experiences of female refugees in addition to males [63]. Previous research findings suggest that it is critical for mental health stigma interventions to improve knowledge about mental health in addition to attitudes [69,71]. Similarly, previous research examining mental health literacy with refugee populations suggests that it is an important barrier to mental health help-seeking [74]. The current study was not designed to evaluate mental health literacy. Further studies investigating the relative contribution of mental health literacy in addition to structural, stigma-related, and refugee-specific barriers would enhance our understanding and call attention to the key strategies to implement when developing interventions to increase help-seeking for mental health.

## 5. Conclusions

With the number of refugees and forcibly displaced people worldwide reaching unprecedented levels it is crucial that we develop and implement interventions aimed at reducing self-stigma related to mental health and encouraging help-seeking amongst these vulnerable individuals. The current study takes a critical first step in identifying the key barriers to mental health help-seeking for these individuals. These findings emphasize mental health stigma and visa status as key barriers to mental health help-seeking for male refugees, and have important implications for service providers and healthcare professionals working with refugee communities.

## Figures and Tables

**Table 1:** Sample characteristics.

	*n*	%	Mean (SD)
**Age**			40.23 (9.94)
**Country of origin**			
Iran	12	11.7	
Iraq	53	51.5	
Sri Lanka	6	5.8	
Syria	25	24.3	
Other	7	6.8	
**Language**			
Arabic	79	76.7	
Farsi	18	17.5	
Tamil	6	5.8	
**Length of time spent in Australia**			
**Visa**			
Secure	67	65	
Insecure	25	24.3	
**Stigma**			
Stigma associated with PTSD			35.56 (9.72)
Stigma associated with help-seeking			25.70 (6.14)
**Practical barriers**			
Not being allowed to work	50	48.5	
Not being able to find work	62	60.2	
Not being able to access English language training	23	22.3	
Not enough money to buy food, pay the rent and bills, or buy necessary clothes	49	47.6	
Difficulty accessing public transport, or not having enough money to use public transport	25	24.3	
Difficulties obtaining financial assistance from Government or Charities	31	30.1	
Difficulties relating to housing (e.g., poor housing conditions, difficulty finding somewhere suitable to live, having to frequently change place of residence)	53	51.5	
**Help-seeking intentions**			
Community			3.27 (1.57)
Family			3.97 (1.62)
Professional			3.87 (1.51)

**Table 2:** Frequencies of trauma exposure based on responses to the Harvard Trauma Questionnaire.

	*n*	%	Mean
Lack of food or water	64	62.10	
Ill health without access to medical care	55	53.4	
Lack of shelter	50	48.5	
Imprisonment	35	34	
Serious injury	51	49.5	
Combat situation (either as a soldier, or as a civilian in an area of conflict)	50	48.5	
Brain washing	24	23.3	
Rape or sexual abuse	16	15.5	
Forced isolation from others	46	44.7	
Being close to death	75	72.8	
Forced separation from family members	41	39.8	
Murder of family or friend	32	31.1	
Unnatural death of family or friend	43	41.7	
Murder of stranger or strangers	33	32	
Lost or kidnapped	44	42.7	
Torture	36	35	
No. of different types of traumatic events experienced or witnessed			7.47

**Table 3:** Summary of indirect effects.

	Est	SE	*p*	95%CI
**PTSD to help-seeking professional**				
via self-stigma for seeking help	−0.16	0.05	0.002	−0.310, −0.040
**PTSD to help-seeking family/friends**				
via self-stigma for PTSD	−0.17	0.05	0.001	−0.332, −0.049
via self-stigma for seeking help	−0.09	0.05	0.060	−0.233, 0.020
via visa status	−0.13	0.05	0.015	−0.286, −0.018
**PTSD to help-seeking community**				
via self-stigma for seeking help	−0.12	0.04	0.007	−0.234, −0.015
via structural barriers	0.09	0.04	0.037	0.008, 0.231
via visa status	−0.13	0.05	0.013	−0.288, −0.022
